# Cre-LoxP-regulated expression of monoclonal antibodies driven by an ovalbumin promoter in primary oviduct cells

**DOI:** 10.1186/1472-6750-11-5

**Published:** 2011-01-14

**Authors:** Isao Oishi, Sungtae Kim, Kyoko Yoshii, Concepcion Rodriguez Esteban, Juan Carlos Izpisua Belmonte

**Affiliations:** 1 Health Research Institute, National Institute of Advanced Industrial Science and Technology, 1-8-31, Midorioka, Ikeda, Osaka 563-8577, Japan; 2 Department of Chemistry, Korea University, Seoul, 136-701, Korea; 3 Gene Expression Laboratory, Salk Institute for Biological Studies, 10010 North Torrey Pines Rd., La Jolla, CA, 92037, USA; 4 Center of Regenerative Medicine in Barcelona, Dr. Aiguader, 88, 08003 Barcelona, Spain

## Abstract

**Background:**

A promoter capable of driving high-level transgene expression in oviduct cells is important for developing transgenic chickens capable of producing therapeutic proteins, including monoclonal antibodies (mAbs), in the whites of laid eggs. Ovalbumin promoters can be used as oviduct-specific regulatory sequences in transgenic chickens, but their promoter activities are not high, according to previous reports.

**Results:**

In this study, while using a previously characterized ovalbumin promoter, we attempted to improve the expression level of mAbs using a Cre/*lox*P-mediated conditional excision system. We constructed a therapeutic mAb expression vector, pBS-DS-hIgG, driven by the CMV and CAG promoters, in which the expression of the heavy and light chains of humanized immunoglobulin G (hIgG) is preceded by two floxed stuffer reporter genes. In the presence of Cre, the stuffer genes were precisely excised and hIgG expression was induced in pBS-DS-hIgG-transfected 293T cells. In chicken oviduct primary culture cells, hIgG was expressed after transfection of pBS-DS-hIgG together with the ovalbumin promoter-driven Cre expression vector. The expression level of hIgG in these cells was increased 40-fold over that induced directly by the ovalbumin promoter. On the other hand, hIgG was not induced by the ovalbumin promoter-driven Cre in chicken embryonic fibroblast cells.

**Conclusions:**

The Cre/*lox*P-based system could significantly increase ovalbumin promoter-driven production of proteins of interest, specifically in oviduct cells. This expression system could be useful for producing therapeutic mAbs at high level using transgenic chickens as bioreactors.

## Background

The market for therapeutic monoclonal antibodies (mAbs) has dramatically expanded over the past decade because of their high clinical efficacy. In the U.S., around 30 mAbs are currently approved for therapeutic use in cancers, autoimmune disorders, and infectious diseases, and the number of available mAb products is predicted to increase [1,2]. Although therapeutic mAbs have become a major class of drugs, their high production cost is a major obstacle. This is mainly due to the use of cultured mammalian cells in the manufacturing of mAbs, which requires a complex industrial bioreactor system. To reduce the cost of mAb production, a more convenient method to replace mammalian cell culture is required.

One alternative method involves generating transgenic farm animals as living bioreactors that produce high-yield therapeutic mAbs in milk or other secretory fluids, such as egg whites. The production of recombinant pharmaceutical proteins has been demonstrated in transgenic animals including sheep, goats, cattle, rabbits, and chickens (reviewed in [3,4]). Among these animals, the use of transgenic chickens as bioreactors is expected to have several advantages, including a shorter timescale for setup, ease of scaling up, and small space requirements (reviewed in [5,6]). Several groups reported the production of therapeutic proteins, such as cytokines, mini-antibodies, and mAbs using transgenic chickens [7-11]. In these transgenic chickens, ubiquitous promoters were used to express the transgenic products; thus, tissue-restricted expression of exogenous proteins was not demonstrated.

Compared to tissue-restricted expression, ubiquitous expression of therapeutic mAbs in transgenic chickens will increase the heterogeneity of oligosaccharide structure of mAbs due to the glycosylation in various type of cells [11,12]. In addition, depending on the antigen recognition, whole-body expression of foreign mAb could be the risk of negatively affecting the development and health of the transgenic chickens. Therefore, oviduct-specific mAb expression is desirable to synthesize mAbs as a component of egg whites. Using chicken ovalbumin promoters, two groups demonstrated oviduct-specific expression of therapeutic proteins in transgenic chickens and secretion of these proteins into the egg whites [12,13] However, expression levels of exogenous proteins in the egg whites driven by ovalbumin promoters were not high (<0.5 mg/ml, egg whites) compared to their expression in the mammalian cell culture bioreactor (1-13 mg/ml, culture media) [13,14]. Thus, a highly efficient oviduct promoter is demanded but such a promoter has not been developed [5].

In an attempt to increase the expression level of therapeutic mAbs in chicken oviduct cells, we developed a Cre-*lox*P-regulated exogenous immunoglobulin G (IgG) expression vector. The vector consists of two tandem expression units, each containing a strong promoter, a fluorescent gene flanked by *lox*P or mutant *lox*P as a stuffer fragment, and the gene for the heavy chain or light chain of humanized IgG (hIgG) encoding the human therapeutic mAb, trastuzumab. Trastuzumab recognizes human epidermal growth facter receptor 2 (HER2), and is clinically used to treat breast cancer. Cre-dependent hIgG induction was observed in mammalian cultured cells as well as laying hen-derived oviduct primary cultured cells following vector transfection. We quantified the expression level of hIgG and observed the 40-fold enhancement of hIgG expression compared to that induced by the ovalbumin promoter as a result of Cre-dependent transcriptional activation.

## Results and Discussion

**Figure 1:**
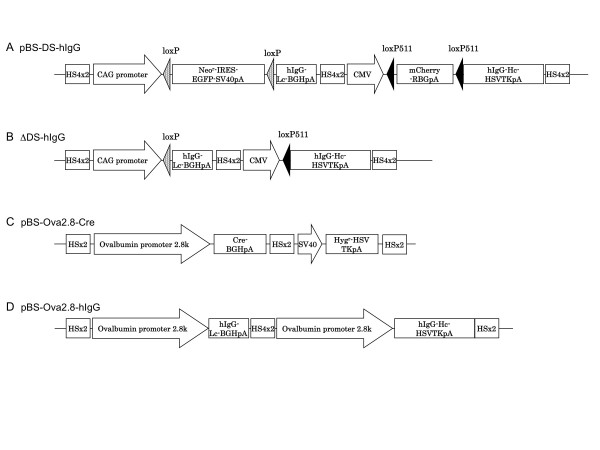
**Schematic representation of the vectors and Cre-mediated recombination**. (A) Structure of pBS-DS-hIgG. Before recombination, neither hIgG light chain nor heavy chain was expressed in the transfected cells because of the insertion of two floxed stuffer sequences located at the 5' end of the hIgG genes. (B) Structure of pBS-DS-hIgG after the deletion of two stuffer sequences (ΔDS-hIgG). Following Cre-mediated recombination of pBS-DS-hIgG, *lox*P- and *lox*P511-flanked stuffer DNA sequences are removed, and then hIgG light and heavy chain expression is driven by the CAG and CMV promoters, respectively. (C) Structure of pBS-Ova2.8-Cre. The vector consists of a 2.8-kb fragment at the 5' end of the ATG codon of chicken ovalbumin (Ova2.8), an oviduct-specific regulatory sequence, and the Cre recombinase gene. (D) Structure of the chicken oviduct-specific hIgG expression vector pBS-Ova2.8-hIgG. In pBS-Ova2.8-hIgG, expression of the hIgG light and heavy chains is individually promoted by the ovalbumin promoter Ova2.8.

To enhance the activity of the ovalbumin promoter and induce efficient production of hIgG in chicken oviduct cells, we utilized a Cre-*lox*P-based conditional gene induction system. The induction system consists of two vectors: pBS-DS-hIgG, an IgG expression vector with two stuffer sequences flanked by *lox*P and modified *lox*P (*lox*P511) sites, and pBS-Ova2.8-Cre, a Cre recombinase expression vector driven by an oviduct-specific ovalbumin promoter contained in a 2.8-kb fragment at the 5' end of the coding sequence of the chicken ovalbumin gene (Ova2.8) (Figure. 1A and 1C). In the absence of Cre recombinase, pBS-DS-hIgG expresses mCherry and EGFP encoded in the stuffer genes, while in the presence of Cre recombinase, the two stuffer DNAs are excised and hIgG light and heavy chain expression commences simultaneously (Figure 1B). Therefore, co-introduction of pBS-Ova2.8-Cre with pBS-DS-hIgG results in oviduct cell-specific Cre-mediated recombination and expression of hIgG driven by two strong promoters, CAG and CMV. In addition to these two vectors, we constructed an ovalbumin promoter-driven hIgG expression vector as a control. This vector consists of two tandem Ova2.8 sequences linked with the light and heavy chains of hIgG (pBS-Ova2.8-hIgG; Figure. 1D).

**Figure 2:**
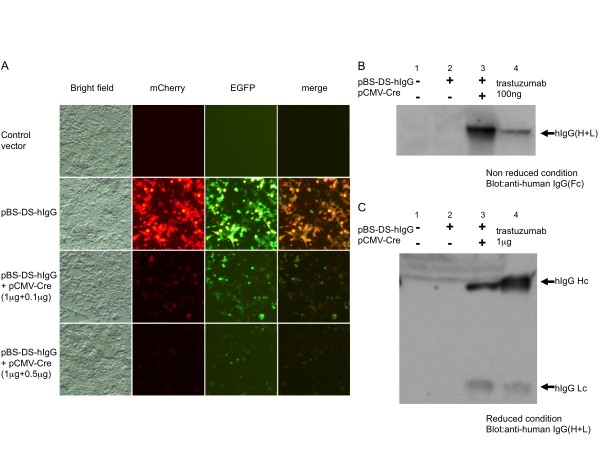
**Cre-mediated hIgG expression in 293T cells**. (A) Cre-mediated reduction of the stuffer gene products in 293T cells. 293T cells were transfected with the control vector (pBS, top row), pBS-DS-hIgG (second row), or co-transfected with pBS-DS-hIgG and a low or high dose of the Cre expression vector (pCMV-Cre, third and bottom rows). Forty-eight hours after transfection, cells were visualized under a fluorescent microscope. Simultaneous expression of EGFP (green) and mCherry (red) was induced from two stuffer genes when cells were transfected only with pBS-DS-hIgG (second row). The expression of EGFP and mCherry were dramatically reduced by Cre expression in 293T cells (third row). The reduction of the stuffer gene expression was Cre-dose dependent (third and bottom rows). (B, C) Cre-dependent hIgG induction in 293T cells. pBS-DS-hIgG was transfected into 293T cells with or without pCMV-Cre. Forty-eight hours after transfection, cell culture supernatants were collected and analyzed by anti-human IgG Fc immunoblotting in the non-reduced condition (B) or by anti-human IgG (H+L) immunoblotting in the reduced condition (C). Expression of hIgG was observed when pBS-DS-hIgG was co-transfected with the Cre expression vector (lane 3, A and C) but not when the IgG expression vector was transfected alone (lane 2, B and C). In the non-reduced condition, hIgG is detectable as a single band of approximately 150 kDa, and in the reduced condition, two bands of approximately 50 and 25 kDa are detected. Trastuzumab was used as a recombinant hIgG control (lane 4, B and C).

Prior to evaluating the vectors in oviduct cells, we analyzed whether pBS-DS-hIgG functioned as expected in cultured mammalian cells. To examine whether both CAG and CMV promoters of pBS-DS-hIgG were active in the same cells, we transfected pBS-DS-hIgG into 293T cells. As shown in Figure. 2A (second row), EGFP (green) and mCherry (red) signals were observed in the transfected cells. Because the signals of EGFP and mCherry were mostly overlapping, both CAG and CMV promoters functioned simultaneously in the transfected cells. Next, we tested whether the two stuffer DNAs in the pBS-DS-hIgG vector could be excised by Cre recombinase. A CMV-driven Cre expression vector (pCMV-Cre) was constructed and co-transfected with the pBS-DS-hIgG vector into 293T cells. Following Cre expression, both EGFP and mCherry fluorescence disappeared in a Cre dose-dependent manner (Figure 2, third and bottom rows). These results suggest that Cre recombinase excised the two floxed stuffer DNAs in the pBS-DS-hIgG vector. Consistent with this idea, hIgG expression was induced by Cre recombinase. 293T cells were transfected with pBS-DS-hIgG alone or in combination with pCMV-Cre, and production of hIgG in the culture media was assessed by immunoblotting. As shown in Figure 2B, hIgG expression was observed when pBS-DS-hIgG was transfected with pCMV-Cre, but not pBS-DS-hIgG alone. In the non-reduced condition, anti-human IgG (Fc) antibody recognized a single band with the same mobility as the recombinant control hIgG protein, trastuzumab (whole antibody). Furthermore, under the reduced condition, anti-human IgG (H+L) antibody detected the heavy and light chains of hIgG in the media of pBS-DS-hIgG and pCMV-Cre co-transfected cells with the same sizes of heavy and light chains of trastuzumab (Figure 2C). These results indicate that Cre-mediated recombination of the pBS-DS-hIgG vector caused the excision of floxed stuffer sequences and the expression of the hIgG complex as a whole antibody similar to trastuzumab for clinical use.

**Figure 3:**
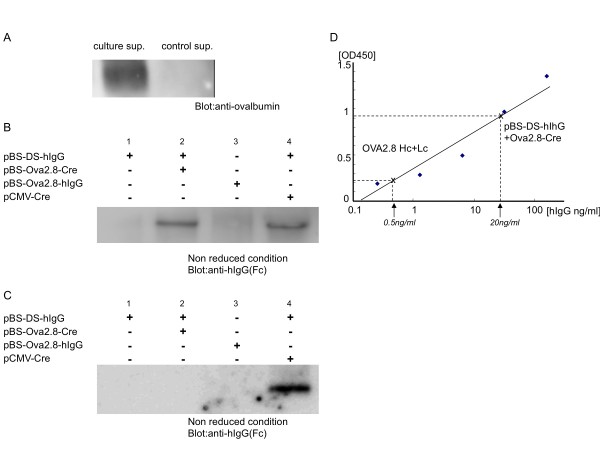
**Cre-mediated hIgG expression in chicken oviduct primary cultured cells**. (A) Expression of ovalbumin protein in oviduct primary cultured cells. Cell culture supernatants were collected 2 days after culture (culture sup.) or at the beginning of cell culture (control sup.) and analyzed by anti-ovalbumin immunoblotting. (B) Expression of hIgG in oviduct primary cultured cells. Cells were isolated from laying hen oviducts as described in Materials and methods. Oviduct primary cultured cells were singly or doubly transfected with the expression vectors, as shown in the panel. The cell culture supernatants were collected and analyzed by anti-human IgG Fc immunoblotting in the non-reduced condition. Obvious expression of hIgG was observed when pBS-DS-hIgG was co-transfected with pBS-Ova2.8-Cre or pCMV-Cre. (C) Expression of hIgG in chicken embryonic fibroblast cells. Fibroblast cells from 9-10 day chicken embryos were transfected with the expression vectors, as shown in the panel. hIgG expression was analyzed as described above. Expression of hIgG was observed when pBS-DS-hIgG was co-transfected with pCMV-Cre but not with pBS-Ova2.8-Cre (D) The induction levels of hIgG were examined by ELISA for human IgG. hIgG is abundant in the media of pBS-DS-hIgG and pBS-Ova2.8-Cre co-transfected oviduct primary culture cells (20 ng/ml) compared to levels in pBS-Ova2.8-hIgG-transfected cells (0.5 ng/ml). Essentially identical results were obtained in three independent experiments.

Next, we examined Cre-mediated hIgG induction by pBS-DS-hIgG transfection in chicken oviduct cells. We isolated cells from the oviducts of laying hens and cultured them. The primary cultured oviduct cells secreted ovalbumin in culture media (Figure 3A), suggesting that ovalbumin expression was activated in the cells. To evaluate the production of hIgG in oviduct cells, the genes were transfected into primary cultured oviduct cells. After two days of culture, secreted hIgG in cell media was analyzed by immunoblotting. As shown in Figure 3B, co-transfection of pBS-DS-hIgG and pCMV-Cre into primary cultured oviduct cells results in abundant production of hIgG in the culture supernatant. hIgG was not detected when pBS-DS-hIgG was transfected alone, but it was clearly observed when pBS-DS-hIgG was co-transfected with pBS-Ova2.8-Cre. Interestingly, obvious hIgG expression was hardly detected in the culture media of oviduct cells transfected with pBS-Ova2.8-hIgG alone. These results indicate that hIgG expression driven by the ovalbumin promoter was enhanced by Cre-*lox*P-based conditional gene induction system in oviduct cells. Consistent with this idea, enhancement of the ovalbumin promoter was not observed in non-oviduct cells. As shown in Figure 3C, hIgG expression was not observed when pBS-DS-hIgG was co-transfected with pBS-Ova2.8-Cre in chicken embryonic fibroblast cells. Taken together, these results suggest that following co-transfection of pBS-DS-hIgG with pBS-Ova2.8-Cre in oviduct primary cultured cells, specific expression of Cre driven by the Ova2.8 promoter excises the stuffer genes in pBS-DS-hIgG and induces strong expression of hIgG in oviduct cells.

The concentration of hIgG in the cell media was analyzed using ELISA (Figure 3D). Consistent with the immunoblotting results, hIgG was more abundant in the culture media of oviduct cells co-transfected with pBS-DS-hIgG and pBS-Ova2.8-Cre (20 ng/ml) than in cells transfected with pBS-Ova2.8-hIgG alone (0.5 ng/ml). Thus, in chicken oviduct cells, pBS-DS-hIgG and pBS-Ova2.8-Cre co-transfection induces a 40-fold greater expression of hIgG than direct induction of hIgG by the ovalbumin regulatory sequence Ova2.8.

In a previous report, Ova2.8 was shown to be the regulatory sequence required for chicken oviduct-restricted expression in vivo [13]. Transgenic hens carrying Ova2.8-driven transgenes specifically express the transgenic products in oviduct cells, and the products are secreted into the egg white (>1.5 mg/egg). However, more efficient production of exogenous protein is required for the commercial use of transgenic chickens as bioreactors to synthesize therapeutic mAbs. In this study, we succeeded in enhancing hIgG expression regulated by Ova2.8 in chicken oviduct cells. Although it is not clear whether the Cre/*lox*P-based gene induction system will induce a 40-fold increase in exogenous gene expression upon its introduction into transgenic chickens, it is expected that strong promoters will induce higer hIgG expression in an oviduct-specific manner.

The size of the transgene unit in pBS-DS-hIgG is over 11 kb. This is larger than the size limitation of the inserted DNA into viral vectors which used to produce transgenic chickens, such as avian leukosis virus vector (size limit 4-5 kb), murine stem cell virus vector (3-4 kb), and lentiviral vector (≈10 kb). Recently, through the use of chicken embryonic stem cells and chicken primordial germ cells, nonviral chicken transgenesis has been developed [12,15,16]. With these new tools, transgenic chickens that carry pBS-DS-hIgG and pBS-Ova2.8-Cre could be established. The creation of transgenic hens carrying these transgenes will demonstrate whether the strategy we have developed can produce living bioreactors that generate therapeutic mAbs at high levels in egg whites.

## Conclusions

It is essential to develop gene expression system that allows high level expression of quality proteins both in research and therapeutic purposes. One promising method is to use chickens as bioreactors where production of proteins of interest is tightly controlled in tissue specific manner to allow accumulation of the gene product only in egg whites. We describe a new method to produce therapeutic mAbs in chicken oviduct cells. This method is based on Cre/*lox*P-mediated conditional excision system, regulated by oviduct specific ovalbumin promoter. In oviduct primary cultured cells, we achieved a 40-fold increase in therapeutic mAb production with this method compared to the mAb induction directly by the ovalbumin promoter. High level Oviduct-specific expression of proteins is required to produce therapeutic mAbs in the egg white. Therefore, this method offers a novel strategy practically useful for producing therapeutic proteins in transgenic chickens.

## Methods

### Transgene construction

The 2.8-kb fragment at the 5' end of the coding sequence of the chicken ovalbumin gene (Ova2.8) was amplified from chicken genomic DNA by PCR and modified to create a new Kozak consensus sequence, essentially as described in a previous report (Table 1) [13]. The insulator element from the chicken β-globin locus was isolated by PCR and two copies of the PCR product were ligated in the same orientation, resulting in a tandem duplication of the insulator (2xHS4) [16]. cDNAs encoding a part of the hIgG mAb trastuzumab light chain (GI:28948772 1-236aa) and heavy chain (GI:28948773 1-105aa) were synthesized and ligated to constant regions of the hIgG light chain (GI:2765423) and heavy chain (GI:2765421), respectively.

**Table 1:** Sequences for PCR primers and synthetic oligo DNAs

**Sequences for PCR primers**	
Ova2.8F	5'-AAGGTACCTTAAGTCCTCAGACTTGGC-3'
Ova2.8R	5'-GCCCCGGGTGAACTCTGAGTTGTCTAG-3'
HS4F	5'-AGGATCCGAAGCAGGCTTTCCTGGAAGG-3'
HS4R	5'-AAGATCTTCAGCCTAAAGCTTTTTCCCCGT-3'
NeoRF	5'-CCGGATCCGATCAAGAGACAGGA-3'
NeoRR	5'-CCAGATCTCAGAAGAACTCGTC-3'
BGHpAF	5'-AGGCCTCGCTGATCAGCCTCG-3'
BGHpAR	5'-AGGTACCGGCCATTACGGCCTGCTATTGTCTTCCCAAT-3'
RGBpAF	5'-CCTCTAGAATTCACTCCTCAGGTGC-3'
RGBpAR	5'-TTGCGGCCGCGGATCCAGGTCGAGGGATCTTCAT-3'
HSVTKpAF	5'-GAATTCTGGGGTTCGAAATGAC-3'
HSVTKpAR	5'-GGATCCTAACCTGAGGCTATGGCA-3'
SV40pF	5'-GATATCTTCAAATATGTATCCGCTCA-3'
SV40pR	5'-GGATCCTCCAAAAAAGCCTCCTCA-3'
Cre-F	5'-GGGTTAACACAACCATGTCCAATTTACTG
Cre-R	5'-TCGGTACCTAATCGCCATCTTC-3'
Sequences for synthetic oligo DNAs	
*loxP*	5'-ATAACTTCGTATAGCATACATTATACGAAGTTAT-3'
*loxP*511	5'-ATAACTTCGTATAGTATACATTATACGAAGTTAT-3'
Em7	5'-GTTGACAATTAATCATCGGCATAGTATATCGGCATAGTATAATACGACAAGGTGAGGAACTAAACC-3'

pBS-DS-hIgG consists of 2xHS4, the CAG promoter of pCAGGS [17], the synthesized *lox*P sequence, the synthesized prokaryotic promoter em7 sequence, the neomycin resistance gene PCR amplified from pEGFP-N1 (Clontech, Mountain View, CA), an IRES sequence followed by the EGFP gene and SV40 polyA of pIRES2-EGFP (Clontech), a second *lox*P sequence, a hIgG light chain, the bovine growth hormone (BGH) polyA PCR fragment amplified from pcDNA3 (Invitrogen, Carlsbad, CA), a second 2xHS4, the CMV promoter of pEGFP-N1, the synthesized *lox*P511 sequence, the mCherry gene (Clontech), a rabbit β-globin (RGB) polyA PCR fragment amplified from pCAGGS, a second *lox*P511 sequence, a hIgG heavy chain, a herpes simplex virus thymidine kinase (HSVTK) polyA sequence amplified from pEGFP-N1, and a third 2xHS4, in this specific order. Each primer sequence for PCR amplification and DNA synthesis is indicated in Table 1. pBS-Ova2.8-Cre consists of 2xHS4, Ova2.8, Cre gene, BGH polyA, a second 2xHS4, the SV40 promoter PCR amplified from pEGFP-N1, the hygromycin resistance gene, HSVTK polyA, and a third 2xHS4, in this specific order (Table 1). pBS-Ova2.8-hIgG consists of 2xHS4, Ova2.8, a hIgG light chain, BGH polyA, another 2xHS4, a second Ova2.8, a hIgG heavy chain, HSVTK polyA, and a third 2xHS4, in this specific order. pCMV-Cre was constructed by ligating the Cre gene into the EcoRI site of pcDNA3 (Invitrogen).

### Cell culture and transfection

293T cells were maintained continuously in DMEM (Invitrogen) supplemented with 10% (v/v) fetal calf serum (FCS). For transfection analysis, cells were plated onto 6-well plates at 3 × 105 cells/well, and 1.5 μg of plasmid was transfected using the FuGene 6 (Roch Diagnostics GmbH, Mannheim, Germany ) transfection reagent according to the manufacturer's protocols. Oviduct cells were dissociated from 18-24-month-old laying hens, as described previously [18,19]. The cells were suspended in DMEM with 10% FCS containing 1 × 10-7 M 17β-estradiol (Nacalai, Tokyo, Japan), 1 × 10-6 M corticosterone (Wako, Osaka, Japan), and 50 ng/ml insulin (Nacalai) and plated onto gelatin-coated 12-well plates. After 24-48 h of culture, cells were grown to 50% confluence and utilized for transfection studies. Before transfection, culture media was replaced with Opti-MEM containing 17β-estradiol, corticosterone, and insulin in the same concentrations as described above. Plasmids were transfected into oviduct primary cultured cells using FuGene6 with 0.5 μg of DNA for 48 h. The total amount of DNA was kept constant with that of the empty vector.

### Immunoblotting analysis

Cell culture supernatants and recombinant hIgG protein (Trastuzumab, Roche) was eluted with non-reducing or reducing Laemmli sample buffer, separated by SDS-PAGE, and transferred to polyvinylidene difluoride membrane filters (Immobilon, Millipore, Bedford, MA). The membranes were immunoblotted with anti-human IgG Fc antibody (I-124, Leinco Technologies, St. Louis, MO) and horseradish peroxidase (HRP)-conjugated anti-human IgG (H+L) antibody (Jackson ImmunoResearch Laboratories, West Grove, PA) for the non-reduced and reduced conditions, respectively. Bound anti-human IgG Fc antibody was visualized with anti-mouse HRP-conjugated antibody using a chemiluminescence reagent (ImmunoStar, WAKO). Bound anti-human IgG (H+L) antibody was directly visualized by the chemiluminescence reagent. To detect ovalbumin expression, anti-ovalbumin antibody (Millipore) was utilized for immunoblotting experiments.

### ELISA

The concentration of human IgG in culture media was measured by a sandwich ELISA with trastuzumab as the standard. Ninety-six-well plates (Nunc Maxisorb, Thermo Fisher Scientific, Rochester, NY) were coated with 100 μl of 10 μg/ml anti-human IgG (I-124) per well overnight at 4°C. Plates were washed twice with 200 μl of Tris-buffered saline (TBS)/0.05% Tween 20 and blocked with 4 × Block Ace (DS Pharma Biomedical, Osaka, Japan) for 1 h at room temperature. After three washes, 100 μl of culture media and trastuzumab standards with 0.02 × Block Ace were added and incubated for 2 h at room temperature. The plates were washed three times and then incubated with 100 μl of 100 ng/ml HRP-conjugated anti-human IgG for 1 h. After three washes, plates were incubated with 100 μl per well of 3,3',5,5'-tetramethylbenzidine solution (TMB One Solution, Promega, Madison, Wisconsin) for 10 min. The reactions were stopped with the addition of 100 μl of 1 M HCl. The optical density of the samples was measured at 450 nm using a microplate reader (Molecular Devices, Sunnyvale, CA)

## Authors' contributions

IO designed, performed the experiment and wrote the manuscript. SK participated in the cell biological studies and drafted the manuscript. KY participated in the vector construction. CRE participated in the cell biological studies. JCIB conceived of the study, participated in its design and coordination of the project and helped to draft the manuscript. All authors read and approved the final manuscript.
